# Liver X Receptor Regulation of Glial Cell Functions in the CNS

**DOI:** 10.3390/biomedicines10092165

**Published:** 2022-09-02

**Authors:** Xiaoyu Song, Wanfu Wu, Margaret Warner, Jan-Åke Gustafsson

**Affiliations:** 1 Center for Nuclear Receptors and Cell Signaling, Department of Biology and Biochemistry, University of Houston, Houston, TX 77204, USA; 2 Department of Biosciences and Nutrition, Karolinska Institutet, 14186 Huddinge, Sweden

**Keywords:** liver X receptors, nuclear receptors, microglia, astrocytes, oligodendrocytes, neurodegenerative diseases

## Abstract

In this review, we discuss the role of liver X receptors (LXRs) in glial cells (microglia, oligodendrocytes and astrocytes) in the central nervous system (CNS). LXRs are oxysterol-activated nuclear receptors that, in adults, regulate genes involved in cholesterol homeostasis, the modulation of inflammatory responses and glutamate homeostasis. The study of LXR knockout mice has revealed that LXRβ plays a key role in maintaining the health of dopaminergic neurons in the substantia nigra, large motor neurons in the spinal cord and retinal ganglion cells in the eye. In the peripheral nervous system (PNS), LXRβ is responsible for the health of the spiral ganglion neurons (SGNs) in the cochlea. In addition, LXRs are essential for the homeostasis of the cerebrospinal fluid (CSF), and in LXRαβ^−/−^ mice, the lateral ventricles are empty and lined with lipid-laden cells. As LXRαβ^−/−^ mice age, lipid vacuoles accumulate in astrocytes surrounding blood vessels. By seven months of age, motor coordination becomes impaired, and there is a loss of motor neurons in the spinal cord of LXRβ^−/−^ mice. During development, migration of neurons in the cortex and cerebellum is retarded in LXRβ^−/−^ mice. Since LXRs are not expressed in dopaminergic or motor neurons in adult mice, the neuroprotective effects of LXRs appear to come from LXRs in glial cells where they are expressed. However, despite the numerous neurological deficits in LXR^−^^/^^−^ rodents, multiple sclerosis has the clear distinction of being the only human neurodegenerative disease in which defective LXR signaling has been identified. In this review, we summarize the regulation and functions of LXRs in glial cells and analyze how targeting LXRs in glial cells might, in the future, be used to treat neurodegenerative diseases and, perhaps, disorders caused by aberrant neuronal migration during development.

## 1. Introduction

Liver X receptors (LXRs) are members of the nuclear receptor supergene family of ligand-activated transcription factors [1]. The family comprises 48 members, and many of these are involved in the physiology and pathology of the central nervous system (CNS). This review focuses on the subfamily called LXRs. There are two members in this family, LXRα (NR1H3) and LXRβ (NR1H2). The first member to be cloned was originally named RLD1 and liver X receptor [2,3], and since then renamed LXRα. LXRβ was discovered in our laboratory [4] and simultaneously and independently in different laboratories [5,6,7], and it was renamed LXRβ due to homology with LXRα. LXRα is mainly expressed in organs involved in lipid metabolism, such as the liver, intestine, adipose tissue, and macrophages. LXRβ is not ubiquitously expressed as has been reported, but it does have a wider tissue distribution being expressed in the immune system, glial cells in the CNS, gall bladder, islets of the pancreas, and prostate epithelium [8,9,10,11,12,13].

LXRs function as heterodimers with retinoid X receptor (RXR) and bind to DNA at response elements called DR4s [1]. DR4s are direct repeats of the half-site sequence 5′-G/AGGTCA-3′, separated by four nucleotides, and are response elements used by the thyroid hormone receptor. Thus, it is not surprising that there is a strong relationship between thyroid hormone and LXR signaling [14,15,16]. Since LXRs regulate cholesterol homeostasis, it is perhaps not surprising that the natural ligands of LXRs are oxygenated forms of cholesterol called oxysterols. These include 22(*R*)-hydroxycholesterol (22-OH), 24(*S*),25-epoxycholesterol, 24(*S*)-hydroxycholesterol (24-OH), 27-hydroxycholesterol (27-OH) and desmosterol, a precursor in the synthesis of cholesterol. The enzyme 24-dehydrocholesterol reductase, which catalyzes the reduction of the delta-24 double bond in desmosterol to cholesterol, is an LXR-regulated gene [17,18]. T0901317 and GW3965 are LXR synthetic ligands that are widely used in research laboratories for in vivo and in vitro studies. Target genes of LXRs, such as apolipoprotein E (ApoE), the ATP binding cassette ABCA1 and ABCG1, are responsible for the modulation of cholesterol homeostasis, while the LXR regulation of glutamine synthetase regulates glutamate at synapses, and the regulation of aquaporins regulates water movement [19,20,21,22,23].

In the adult mouse CNS, LXRs are expressed in microglia [24], astrocytes [25] and oligodendrocytes [26]. LXRs are not detected in neurons of the adult mouse brain. However, LXRs are expressed in cultured neurons and glial cells isolated from fetal brains [27]. Thus, LXRs appear to have functions in neurons during fetal life that are lost in adults.

Several previous reviews have covered the role of LXRs in cholesterol metabolism and lipid signaling, as well as the regulation of LXRs in the CNS and peripheral nervous system (PNS) diseases [20,28,29,30,31]. In this review, we focus on the regulatory roles of LXRs in glial cells and discuss glia–neuron interactions as novel mechanisms through which LXRs exert neuroprotective effects.

## 2. LXRs and Microglia

Microglia, a major cell population in the CNS, are key regulators of inflammatory responses. According to their function in immune responses, microglia have been designated as M1 and M2 types. M1 microglia contribute to the development of inflammation by producing pro-inflammatory cytokines, while M2 microglia exert anti-inflammatory effects by enhancing the expression of anti-inflammatory cytokines and also exhibit phagocytic activity to promote the removal of cellular debris and misfolded proteins.

**Figure 1:**
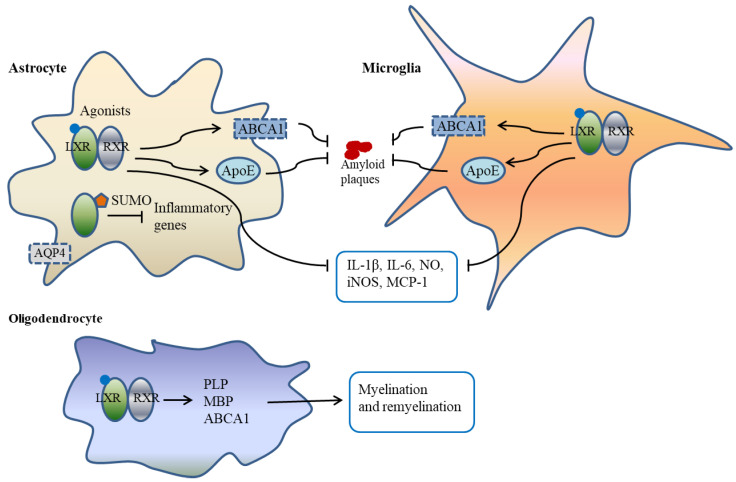
Schematic diagram illustrating the role of LXRs in glial cells. In microglia and astrocytes, LXRs decrease the production of pro-inflammatory mediators such as IL-1β, IL-6, NO, iNOS and MCP-1. In addition, LXRs regulate the expression of ApoE and ABCA1, proteins that inhibit amyloid plaque deposition by promoting phagocytosis. In astrocytes, LXR ligands trigger post-translational modification by SUMO allowing LXRs to enter the transrepression pathway to suppress inflammatory gene expression. LXRs regulate the expression of AQP4 on the astrocytic end feet and participate in the regulation of water transport at the blood–brain barrier. In oligodendrocytes, LXRs regulate the expression of myelinating genes such as PLP, MBP and ABCA1, thereby participating in the regulation of myelination and remyelination. Abbreviations: ABCA1, ATP binding cassette subfamily A member 1; ABCG1, ATP binding cassette subfamily G member 1; ApoE, apolipoprotein E; AQP4, aquaporin 4; RXR, retinoid X receptor; IL-1β, interleukin-1β; IL-6, interleukin-6; iNOS, inducible nitric oxide synthases; LXRs, liver X receptors; MBP, myelin basic protein; MCP-1, monocyte chemoattractant protein-1; NO, nitric oxide; PLP, proteolipid protein; SUMO, small ubiquitin-like modifier.

LXRs have potent anti-inflammatory activities in the CNS mediated by their effects on microglia. The overactivation of microglia and astrocytes triggers the release of pro-inflammatory mediators, such as interleukin-1β (IL-1β), interleukin-6 (IL-6), tumor necrosis factor α (TNFα), nitric oxide (NO), cyclooxygenase-2 (COX-2), and expression of inducible nitric oxide synthases (iNOS). These inflammatory responses of microglia contribute to neuronal death in diseases such as Alzheimer′s disease (AD), Parkinson′s disease (PD), amyotrophic lateral sclerosis (ALS), multiple sclerosis (MS) and retinal degeneration [32,33] (Figure 1).

LXRs are involved in the regulation of microglial functions and neuroinflammation [34,35,36] and LXR agonists T0901317 and GW3965 inhibit the production of NO, IL-1β, IL-6 and monocyte chemoattractant protein-1 (MCP-1) in microglia and astrocytes [37,38].

## 3. LXRs and Oligodendrocytes

Oligodendrocytes are responsible for myelinating neuronal axons, and cholesterol synthesis and transportation in oligodendrocytes are essential for normal myelination and are key for remyelination in demyelinating diseases such as MS.

Several nuclear receptors regulate oligodendrocyte differentiation and myelination [39]. LXRs differentially affect the mRNA amounts of myelin genes in myelin-rich tissues, such as spinal cord, corpus callosum, optic nerve and cerebellum [40]. Ligands of LXRs affect the mRNA level of myelin-related genes, proteolipid protein (PLP) and myelin basic protein (MBP) [41]. Activation of LXRs also promotes oligodendrocyte maturation [41]. In the adult rodent CNS, oligodendrocyte progenitor cells proliferate, migrate, and differentiate into myelinating oligodendrocytes [42]. Oligodendrocyte precursor cells express both the platelet-derived growth factor receptor α (PDGFRα) and the chondroitin sulfate proteoglycan NG2 [43]. PDGFRα is an LXR-regulated gene and its regulation by LXRs may be one mechanism through which LXRs regulate the number of oligodendrocytes in the CNS.

## 4. LXRs and Astrocytes

Astrocytes are the most abundant glial cell type in the CNS and are involved in many aspects of brain physiology and pathology. As shown in Table 1, loss of LXRβ leads to the activation of astrocytes in the spinal cord, substantia nigra, retina, optic and cochlear nerves [44,45,46,47,48,49,50]. The water channel aquaporin 4 (AQP4) is an LXR-regulated gene [23]. It is expressed in the astrocytic end feet and ependymal cells, where it regulates the homeostasis of the cerebrospinal fluid (CSF). Astroglial water transport supports CSF flux into the parenchyma and facilitates bulk interstitial fluid solute clearance from the parenchyma [51]. In LXRαβ^−/−^ mice, there is a severe defect in the maintenance of CSF resulting in occlusion of the lateral ventricles and degeneration of the choroid plexus [23,30].

LXR agonists inhibited the expression of NO, IL-1β, IL-6 and MCP1 from LPS-treated astrocytes [37]. Previous studies have suggested that LXR agonists inhibit astrocyte and microglia activation, thereby inhibiting neuroinflammation and exerting a protective effect in several different animal models of AD and PD [47,60,61,62,63,64]. Cholesterol synthesis and clearance by astrocytes are tightly regulated to maintain homeostasis within the brain, and regulation by LXRs of ApoE expression, secretion and cholesterol homeostasis is essential for the beneficial effects of astrocytes [61,65,66].

LXRs suppress the expression of inflammatory genes in a context-specific manner. Previous studies have shown that in macrophages and hepatocytes, LXR ligands trigger post-translational modification by small ubiquitin-like modifier (SUMO), allowing LXRs to enter the transrepression pathway [67,68]. Additionally, SUMOylation is required for the suppression of signal transducer and activator of transcription 1 (STAT1)-dependent inflammatory responses from LXRs in interferon-γ (IFN-γ)-stimulated brain astrocytes [69]. It has been suggested that a small heterodimer partner mediates the anti-inflammatory actions of LXRs through differential regulation of receptor SUMOylation specifically in astrocytes [25], thereby revealing potential avenues for therapeutic development in diseases associated with brain inflammation (Figure 1).

## 5. LXRs in Multiple Sclerosis

MS is an inflammatory demyelinating disease whose precise etiology is not clear, although several factors, including genetic and environmental factors, have been implicated [70]. In the active phase of the disease, pro-inflammatory microglia phagocytize myelin debris, but prolonged inflammation causes damage, and, in order for lesions to heal, microglia have to switch from a pro-inflammatory state (M1) to a repair mode (M2). LXRs play two key roles in MS. They modulate inflammation, and they stimulate oligodendrocyte repair [71,72]. Mailleux et al. have shown that the processing of myelin by phagocytes releases LXR ligands and that LXRs are upregulated in phagocytic microglia in MS lesions [73]. In addition, phagocytic microglia synthesize desmosterol, which is an LXR agonist [74]. Thus, LXRs are involved in inflammation in MS, and LXR ligands may, in the future, be used to dampen inflammation in MS.

## 6. LXRs in Alzheimer′s Disease

AD is an age-related neurodegenerative disease characterized by extracellular plaques composed of amyloid beta (Aβ). Both increased synthesis and inefficient clearance of Aβ contribute to plaque buildup, and inhibiting Aβ formation or promoting its clearance is a target for the treatment of this disease [75]. There are several mouse models and cell lines that are used extensively to study AD. Some overexpress APP and some express the human APOE4 variant, which is associated with AD [76]. In the APP/PS1 transgenic mouse, a mouse model in which there is of buildup Aβ in the brain, loss of LXRα or LXRβ results in increased amyloid plaque burden [52]. In BV2 cells (immortalized mouse microglial cells), GW3965 regulates inflammatory responses and increases the ability to phagocytize Aβ fibrils. In APP23 mice, LXR-agonist treatment attenuates Aβ deposition and facilitates its clearance [77]. The inhibition of microglia and astrocyte activation is one of the main mechanisms by which LXR agonists exert protection in different AD models [62,63].

Another beneficial function of LXR activation is the induction of the release of ApoE, which is critical for the ability of glial cells to remove Aβ [60,61]. ApoE carries lipids in the brain in the form of lipoproteins and promotes the proteolytic degradation of Aβ [78]. TREM2 (triggering receptor expressed on myeloid cells 2) is expressed in microglia where it upregulates ApoE and other damage-associated microglia genes. Loss of either TREM2 or ApoE leads to dysregulated cholesterol transportation and metabolism in microglia [79].

One interesting LXR-regulated gene is cytochrome P450 46A1 (CYP46A1). It is the enzyme responsible for the synthesis of 24-OH, which is the main excretory pathway of cholesterol in the CNS and is a pharmacological target for AD due to its important role in cholesterol homeostasis [80]. In support of an important role of cholesterol homeostasis in AD, Combarros et al. have shown in a case–control study that an intron 2 CYP46 T/C gene polymorphism is associated with increased brain Aβ load and a higher risk of AD [81].

We have found that there is spontaneous build-up of Aβ around the ventricles in LXRβ^−/−^ mice. This was not accompanied by an activation of microglia or astrogliosis or an increase in neuronal apoptosis. Astroglial-mediated interstitial fluid bulk flow, facilitated by astroglial AQP4 channels and named the glymphatic system, contributes to a larger portion of extracellular Aβ clearance [75,82]. Loss of perivascular AQP4 localization impairs glymphatic exchange and promotes Aβ plaque formation in mice [38]. The regulation of LXRs in Aβ accumulation and clearance systems in the brain (e.g., interstitial fluid bulk clearance, perivascular glymphatic and lymphatic systems) remains largely unknown.

## 7. LXRs in Parkinson′s Disease

Although in LXRβ knockout mice, there is a loss of dopaminergic neurons, there is so far no association between LXRs and PD in humans. MPTP (1-methyl-4-phenyl-1,2,3,6-tetrahydropyridine), a chemical originally found as a contaminant in street drugs, causes the loss of dopaminergic neurons. In rodents, the ablation of LXRβ aggravates the MPTP-induced loss of dopaminergic neurons and activation of microglia and astrocytes in the substantia nigra. The LXR agonists GW3965 and T0901317 reduce the activation of glial cells [47], suppress inflammatory responses, attenuate the activation of microglia and protect dopaminergic neurons from MPTP-induced impairment [64]. T0901317 and GW4965 also exert protective effects by inhibiting microglial activation and neuroinflammation in experimental autoimmune encephalomyelitis (EAE), experimental intracerebral hemorrhage and sleep-deprived cognitive impairment models [83,84,85].

## 8. LXRs in Ocular Neurodegenerative Diseases

Aged mice lacking LXRs develop isoform-dependent ocular pathologies. Loss of LXRs leads to retinal vascular injury and the formation of acellular capillaries similar to diabetic retinopathy [50]. We have reported that, in LXRβ knockout mice, there is inflammation of the optic nerve and a loss of ganglion cells from the retina. This is accompanied by increased activation of microglia, loss of AQP4 in astrocytes, and a decrease in oligodendrocytes and glutamine synthetase in the optic nerve [48]. Loss of LXRα in mouse eyes results in a pathobiology resembling age-related macular degeneration (AMD) [86]. Inactivation of CYP46A1 causes microglia/macrophage activation and a retinal phenotype typical of diabetic retinopathy [87], strongly supporting the idea that defective cholesterol metabolism is involved in retinal dysfunction. LXRs have great potential in the treatment of retinal degeneration such as AMD by regulating microglial activation and the inflammatory response [88]. N, N-dimethyl-3β-hydroxy-cholenamide, a selective LXR agonist, corrected retinal dysfunction in type 2 diabetes [89]. GW3965 treatment reduced activated microglia and inflammatory monocytes in the retina of streptozotocin-diabetic DBA/2J high-fat Western diet mice [50]. T0901317 treatment decreased the activation of microglia and gliosis of Müller cells, and decreased the expression levels of IL-6, iNOS and COX-2 [90]. In addition, the activation of LXRs restores reverse cholesterol transportation, prevents inflammation and reduces pro-inflammatory macrophage activity in several retinal degeneration models [91,92,93,94]. A clinical trial on “LXR as a novel therapeutic target in diabetic retinopathy” (NCT03403686) is ongoing, and there is hope that novel pharmaceuticals will soon be available for clinical use in the treatment of retinal disorders.

## 9. Concluding Remarks

LXRs regulate glial cell functions and play an important role in neurodegenerative diseases.

The synthetic LXR ligands available today are associated with side effects such as hypertriglyceridemia and hepatic steatosis that limit their clinical application. Some new drug delivery systems, such as the DMHCA (a LXR partial agonist) polymer therapeutic approach [95], and phytosterols, such as sargassum fusiforme [96], which work without increasing cholesterol/triglyceride levels, are very promising in the treatment of neurodegenerative diseases.

The establishment of a glial cell (microglia, astrocytes, oligodendrocytes)-specific LXR knockout model will facilitate the identification of the glial cells in the CNS whose functions are changed by the loss of LXRs, as well as an understanding of how LXRs exert a neuroprotective effect. Studies of cell-type-specific LXR knockouts are already underway, and we have made some exciting findings: loss of LXRβ in astrocytes leads to anxiety-like behaviors [97], and LXRβ deficiency affects the inflammatory features of microglia in vitro. However, these changes do not underlie the reduced EAE disease severity in whole-body LXRβ knockout mice [24]. We look forward to more experimental findings in this regard. Additionally, although a large number of studies have shown that LXRs play multiple important roles in rodents, so far in clinical neurodegenerative diseases, only in multiple sclerosis has an association been made with LXRs. The different genomic and physiological functions of LXRs in humans and rodents cannot be ignored [98]. Therefore, more studies on LXR signaling in humans or nonhuman primates are needed.

## Figures and Tables

**Table 1:** Summary of neurological phenotypes resulting from LXR deletion.

Knockout	Phenotype	Related Diseases	Changes in Glial Cell Function	Refs.
LXRβ^−/−^	Loss of motor neurons in the spinal cord.	Amyotrophic lateral sclerosis	Activation of astrocytes, accumulate cholesterol and progressive inflammation.	[44,45,46]
	Loss of either LXRα or β in APP/PS1 mice results in increased amyloid plaque load.	Alzheimer′s disease	GW3965 regulates inflammatory responses and phagocytic ability of Aβ fibrils.	[44,45,46,52]
	Late-generated neocortical neurons do not migrate.	Psychiatric disorders	Delayed oligodendrocyte differentiation and maturation.	[43,53,54,55]
	In a MPTP model, loss of dopaminergic neurons in the substantia nigra.	Parkinson′s disease	Increased activation of microglia and astrocytes in the substantia nigra.	[47]
	Loss of retinal ganglion cells.	Optic neuritis	Loss of AQP4 in astrocytes and increased activation of microglia in the optic nerve.	[44,45,46,48]
	Loss of spiral ganglion neurons (peripheral nervous system).	Age-related hearing loss	Increased activation of microglia in the cochlear nerve, activation of macrophages.	[49]
LXRαβ^−/−^	Occlusion of the lateral ventricles and degeneration of the cells of the choroid plexus.	Cytotoxic brain edema	AQP4 expression was increased in astrocytes.	[23,30]
	Loss of dopaminergic neurogenesis in the ventral midbrain of all LXR-null mice.	Parkinson′s disease		[56]
	Reduced thickness of myelin sheaths, enhanced anion superoxide production and lipid oxidization in the sciatic nerves (peripheral nervous system).	Demyelinating diseases	Involvement of Schwann cell function.	[57,58,59]
	Retinal vascular injury and formation of acellular capillaries.	Diabetic retinopathy	Activated glial cells and inflammatory monocytes were reduced in retinas from GW3965-treated animals.	[50]
	Altered motor coordination and spatial learning, thinner myelin sheaths.	Demyelinating diseases	LXR agonists promote oligodendrocytes maturation and remyelination.	[41]

## Data Availability

Not applicable.

## Abbreviations

22-OH: 22(R)-hydroxycholesterol; 24-OH, 24(S)-hydroxycholesterol; 27-OH, 27-hydroxycholesterol; Aβ, amyloid beta; ABCA1, ATP binding cassette subfamily A member 1; ABCG1, ATP binding cassette subfamily G member 1; AD, Alzheimer’s disease; ALS, amyotrophic lateral sclerosis; AMD, age-related macular degeneration; ApoE, apolipoprotein E; AQP4, aquaporin 4; CNS, central nervous system; COX-2, cyclooxygenase-2; CSF, cerebrospinal fluid; CYP46A1, cytochrome P450 46A1; EAE, experimental autoimmune encephalomyelitis; ER, endoplasmic reticulum; RXR, retinoid X receptor; IFN-γ, interferon-γ; IL-1β, interleukin-1β; IL-6, interleukin-6; iNOS, inducible nitric oxide synthases; LPS, lipopolysaccharide; LXRs, liver X receptors; MBP, myelin basic protein; MCP-1, monocyte chemoattractant protein-1; MPTP, 1-methyl-4-phenyl-1,2,3,6-tetrahydropyridine; MS, multiple sclerosis; NO, nitric oxide; PD, Parkinson’s disease; PDGFRα, platelet-derived growth factor receptor alpha; PLP, proteolipid protein; PNS, peripheral nervous system; SGNs, spiral ganglion neurons; STAT1, signal transducer and activator of transcription 1; SUMO, small ubiquitin-like modifier; TGFβ, transforming growth factor β; TNFα, tumor necrosis factor α; TREM2, triggering receptor expressed on myeloid cells 2.
